# Sarcoidosis lymphoma syndrome - the value of PET-CT in the diagnosis

**DOI:** 10.1186/1477-7819-11-235

**Published:** 2013-09-18

**Authors:** Adrian Kis, Noemi Eszes, Lilla Tamasi, Gyorgy Losonczy, Attila Csekeo, Judit Csomor, Veronika Muller

**Affiliations:** 1 Department of Pulmonology, Semmelweis University, 1125, Diós árok 1/C, Budapest, Hungary; 2 Koranyi National Institute for Tuberculosis and Pulmonology, 1121, Pihenő Street 1, Budapest, Hungary; 3 1st Department of Pathology and Experimental Cancer Research, Semmelweis University, 1085, Üllői Street 26, Budapest, Hungary

**Keywords:** Sarcoidosis, Malignancy, Sarcoidosis lymphoma syndrome, PET-CT

## Abstract

We report a 52-year-old patient who developed B-cell non-Hodgkin’s lymphoma subsequent to sarcoidosis. Sarcoidosis was diagnosed 16 years ago and remained asymptomatic for 14 years after steroid treatment. She presented with new symptoms of arthralgia, photosensitivity, butterfly erythema, autoimmune antibodies (ANA, chromatin positivity) associated with progression of the known left upper lobe lesion on the chest X-ray suggesting primary autoimmune disease (systemic lupus erythematosus). As steroid treatment was not effective, we started bolus cyclophosphamide therapy after which progression was seen on the chest X-ray. Computed tomography (CT)-guided needle biopsy confirmed malignancy of indefinable origin. Despite of the well-known fluorodeoxyglucose (FDG) avidity in active sarcoidosis, a FDG-positron emission tomography (PET) scan was performed to stage the primary tumour. Intensive FDG uptake was detected in the affected lung segment, with moderate uptake in mediastinal lymph nodes. The patient underwent left upper lobectomy. The histology showed pulmonary mucosa-associated lymphoma (bronchus-associated lymphoid tissue (BALT) lymphoma) in the lung tissue, while only sarcoidosis was present in the mediastinal lymph nodes. Bone marrow biopsy was negative.

The association between sarcoidosis and lymphoma is known as sarcoidosis lymphoma syndrome, which is a rare disease. PET-CT was helpful in the differentiation of sarcoidosis and malignancy in this patient. It is important to be aware of the risk of lymphoma in sarcoidosis and FDG-PET, used for adequate purpose, can help the diagnosis.

## Background

Sarcoidosis is a chronic granulomatous inflammatory multisystem disorder of unknown origin. The association between sarcoidosis and malignancy - particularly lymphoproliferative disease, such as non-Hodgkin’s lymphoma - has been previously described [1-3]. In sarcoidosis lymphoma syndrome, sarcoidosis is followed by the development of the lymphoproliferative disorder [4]. Higher fluorodeoxyglucose (FDG)-uptake may be seen in both diseases; therefore, positron emission tomography-computed tomography (PET-CT) cannot exclude or prove the presence of malignancy in sarcoidosis patients. In our case report of sarcoidosis lymphoma syndrome, we demonstrate how PET-CT could help within the decision making process.

## Case presentation

**Figure 1:**
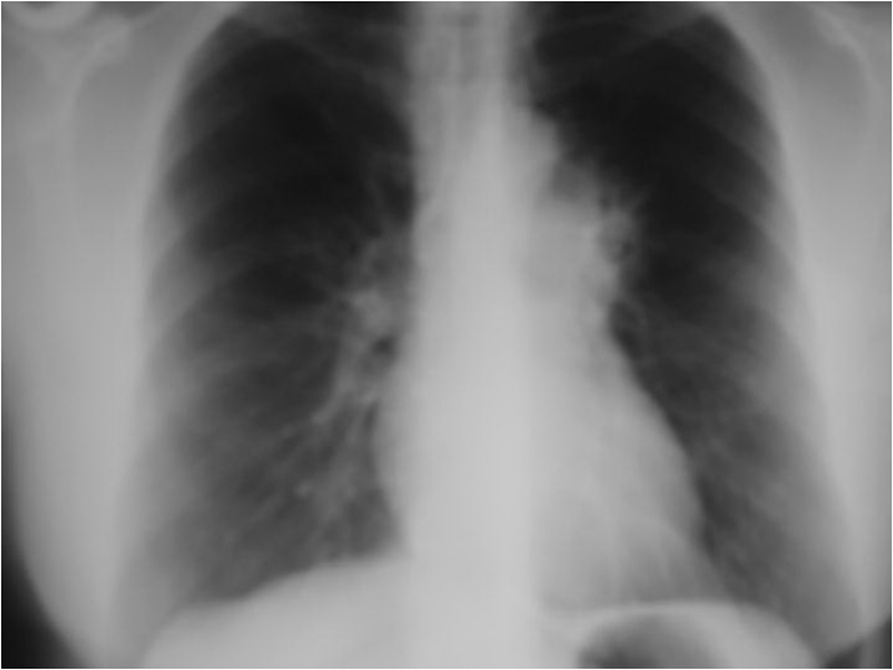
**Chest X-ray of the patient.** After 14 asymptomatic years, new symptoms occurred. The chest X-ray showed progression of the known left upper lobe lesion.

**Figure 2:**
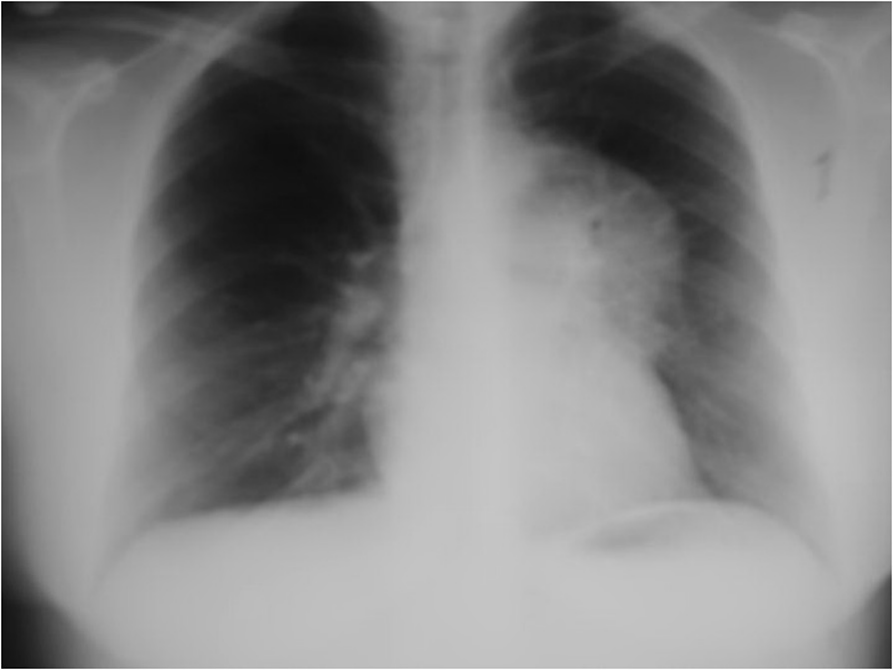
**Repeat chest X-ray.** Repeat chest X-ray showed rapid and significant worsening of the left upper lobe lesion.

**Figure 3:**
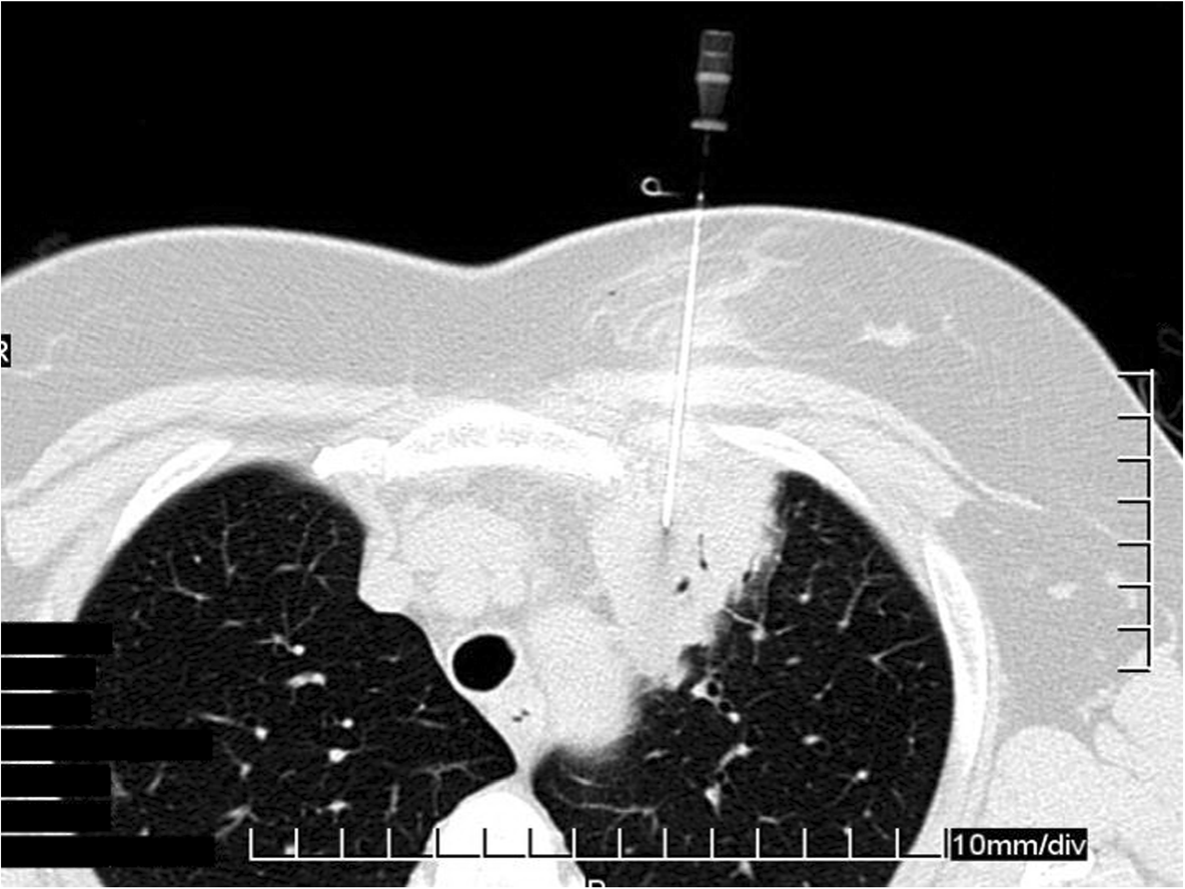
Computed tomography (CT) guided needle biopsy of the left upper lobe lesion.

The 52-year-old female patient first presented in 1992 with erythema nodosum, alopecia and a dry cough. Chest X-ray and computed tomography (CT) showed bilateral hilar lymphadenopathy and lung parenchyma involvement. Histology confirmed sarcoidosis, which was treated with systemic steroids for six months, after which persisting diabetes mellitus developed. After 14 asymptomatic years without immunosuppressive therapy, she was referred to our department with symptoms of dry cough, snoring and mild anaemia. On the chest X-ray, progression of the known left upper lobe lesion was noted (Figure 1). As primarily progression of sarcoidosis was suspected, steroid treatment was restarted. During the immunosuppressive therapy arthralgia, butterfly erythema, photosensitivity appeared, associated with anti-nuclear and anti-chromatin antibody positivity. A lung function test revealed decreased diffusion capacity of carbon monoxide (CO). According to the American College of Rheumatology’s criteria, an additional diagnosis of systemic lupus erythematosus (SLE) was established. Due to ineffective response to steroid treatment, bolus cyclophosphamide (500 mg/m^2^ monthly) therapy was instituted. Repeated chest X-ray showed rapid and significant worsening of the left upper lobe lesion following the first dose (Figure 2). Beside the left upper lobe lesion, enlarged mediastinal lymph nodes and a small nodular lesion in the left lower lobe (segment 10) were noted on the chest CT. Histology of the transbronchial biopsy from the left upper lobe showed nonspecific lymphocytic inflammation. CT-guided needle aspiration of the left upper lobe lesion was performed to get proper diagnosis (Figure 3). Cytology confirmed malignancy of indefinable, but possible renal origin. Abdominal ultrasound and CT scan were negative.

**Figure 4:**
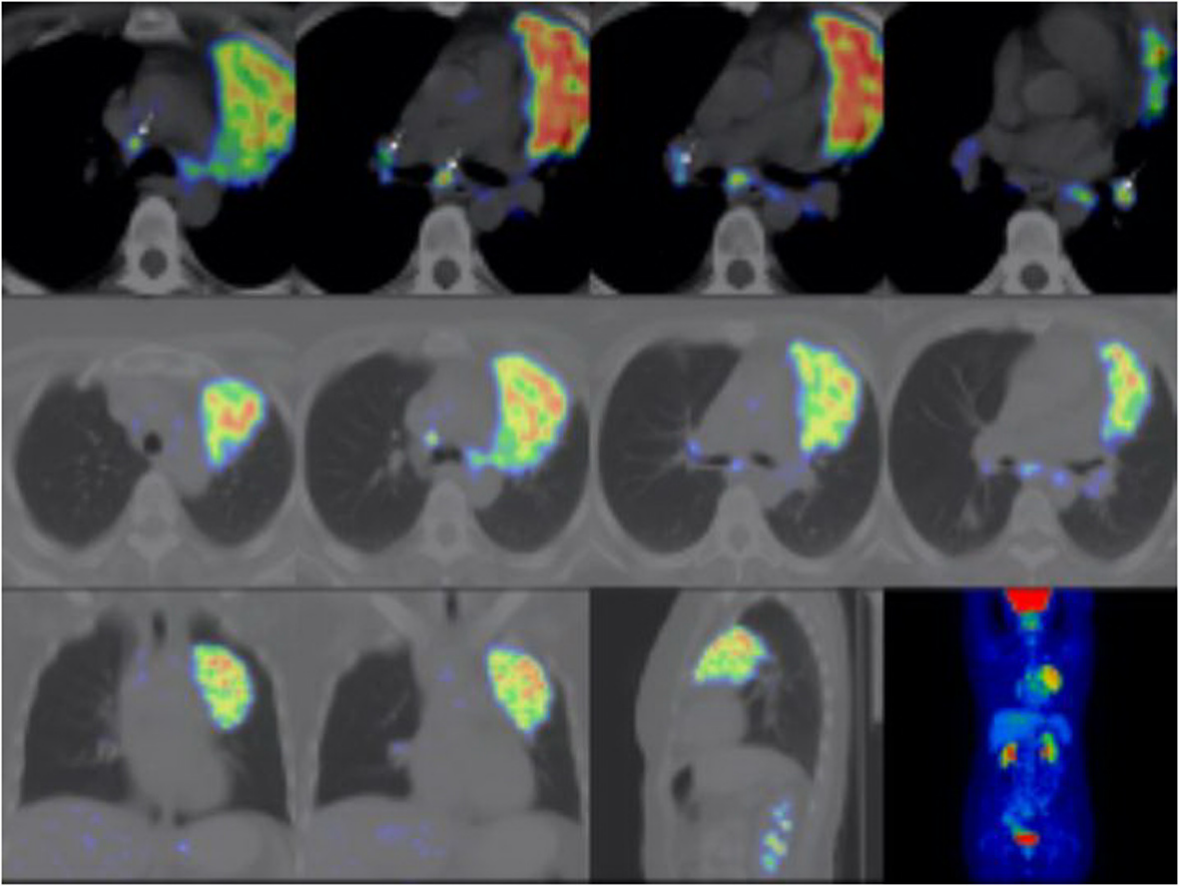
Fluorodeoxyglucose (FDG)-positron emission tomography (PET) scan.

To localize the primary tumour and its stage, FDG-PET-CT scan was made, knowing that active sarcoidosis is associated with increased FDG uptake. Intensive FDG uptake was detected in the affected lung segment, with only moderate uptake in mediastinal lymph nodes (Figure 4). As the nodule in the left lower lobe showed moderate FDG uptake similar to mediastinal lymph nodes, it was suggested to represent sarcoidosis. Taking into consideration the very intensive FDG uptake and cytology confirmed malignancy of the left upper lobe, our surgical team came to the decision of performing a left upper lobectomy and extensive lymph node sampling.

**Figure 5:**
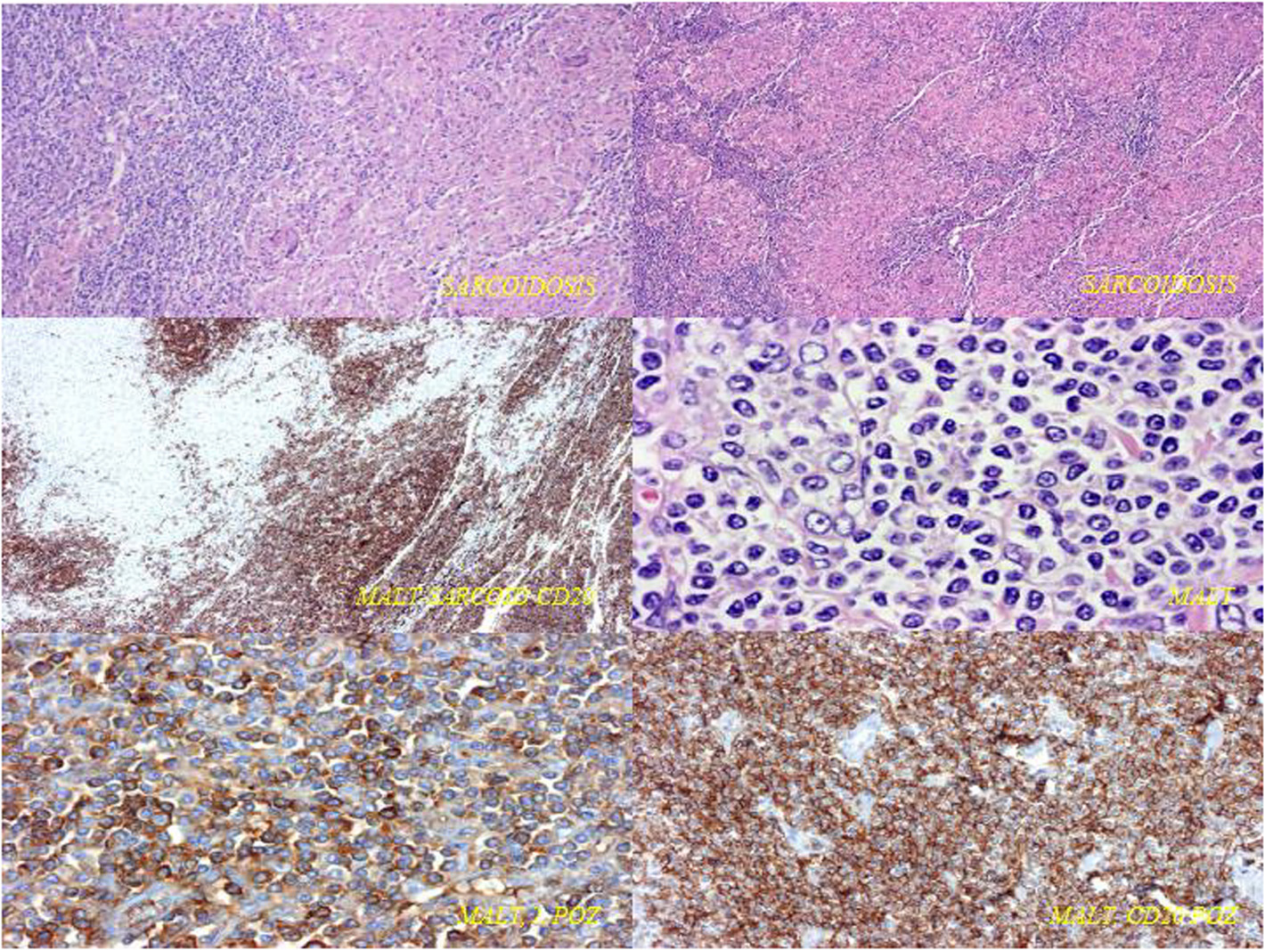
**Immunophenotyping of pulmonary mucosa-associated lymphoma.** B cell lineage phenotype was demonstrated: CD20, BCL2 and lambda light chain positivity on the cell surface with CD10 and CD5 negativity.

**Figure 6:**
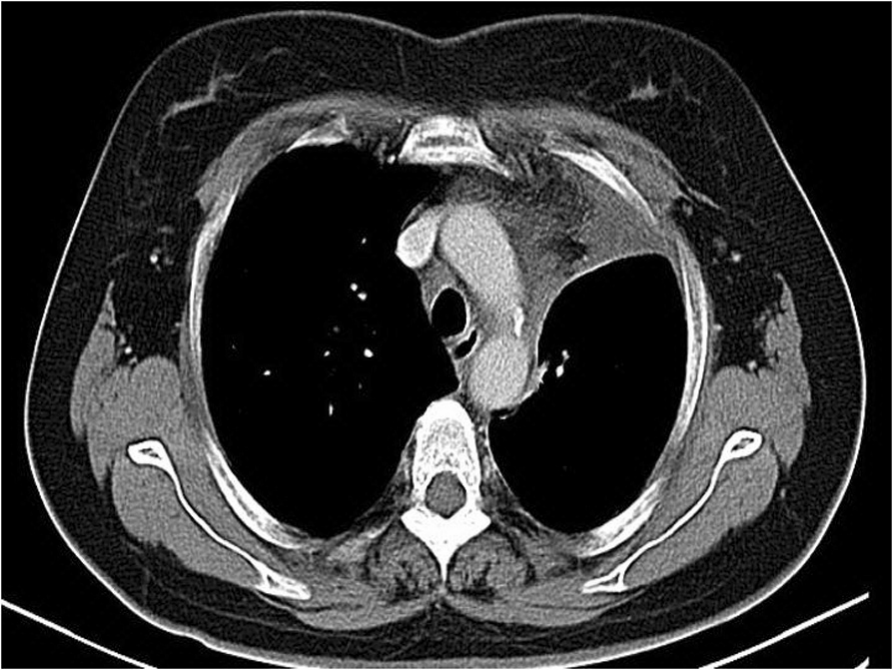
**Repeat chest computed tomography (CT) scan one year after surgery.** One-year follow-up showed no progression of the diseases.

**Figure 7:**
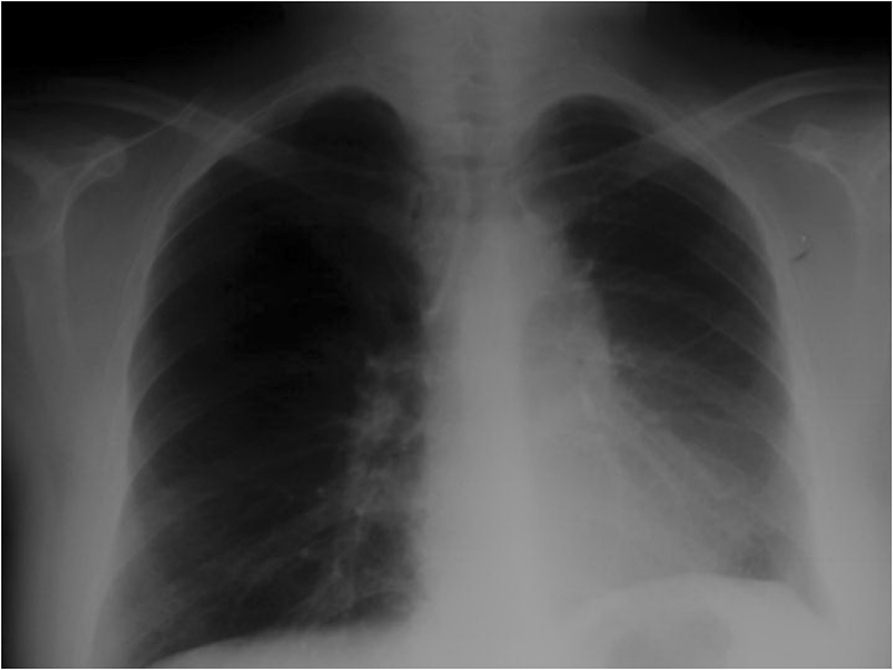
**Repeat chest X-ray one year after surgery.** One-year follow-up showed no progression of the diseases.

Postoperative histology showed pulmonary mucosa-associated lymphoma (bronchus associated lymphoid tissue (BALT) lymphoma) in the left upper lobe lung tissue, while only sarcoidosis was present in the mediastinal lymph nodes. Immunophenotyping demonstrated a B cell lineage phenotype: CD20, BCL2 and lambda light chain positivity on the cell surface, with CD10 and CD5 negativity (Figure 5). The tumour was confined only to the lung parenchyma of the left upper lobe; haematological examinations, including bone marrow biopsy, were negative, confirming the diagnosis of stage I BALT lymphoma. After surgery the patient received only respiratory rehabilitation. Three-year follow-up with regular chest-abdominal CT scans showed no progression or recurrence of the diseases (Figures 6 and 7).

Sarcoidosis is a multisystem granulomatous disorder of uncertain origin. The diagnosis of sarcoidosis is based on clinical, radiological, biochemical and histological findings. These findings are erythema nodosum, lymphopenia, hypergammaglobulinemia, anergy, hypercalcemia and elevated angiotensin-converting enzyme. The non-caseating granulomas are B-cell negative [1]. The results should be carefully interpreted, because sarcoid-like granulomas can be found in many other disorders, such as chronic inflammatory, infectious and neoplastic diseases [2]. Incidence is influenced by age, race and geographical location. The subacute form diagnosed in most patients under the age of 30 affects mainly intrathoracic organs and the duration of the disease is generally less than two years. The chronic form begins more often over the age of 40 and might involve extrathoracic organs [1].

Brincker and Wilbek have matched all patients who had been diagnosed with respiratory sarcoidosis during the period 1962 to 1971 against the data of the Cancer Registry. About 1.5 times higher incidence of cancer was seen in sarcoidosis patients. Lung cancer occurred 3 times and malignant lymphoma 11.5 times more frequently than in the control population [3]. In a later study, Brincker found three features of malignancies following sarcoidosis based on the analysis of 29 case reports in the literature [1]: the median age of the patients was 41 years at the diagnosis of sarcoidosis, which indicates a late onset, and is more often associated with a chronic type [2]. Lymphoproliferative disease (LD) has occurred with a median interval of 24 months after the diagnosis of sarcoidosis [3]. Hodgkin’s disease was diagnosed more frequently than other types of lymphomas. Further analysis of 17 cases revealed that LD developed 5.5 times more frequently than expected, which may suggest that the chronic active type of sarcoidosis is a predisposing factor for LD. Brincker introduced the term sarcoidosis lymphoma syndrome for this specific condition [4]. In a retrospective cohort study Askling and his colleagues assessed the risk for malignancy in sarcoidosis. They linked a sarcoidosis incidence study (Uppsala Cohort) and patients identified with sarcoidosis in the Swedish Inpatient Register to population-based registers (Cancer Register, Register of Causes of Death, Register of Total Population). Significantly increased risk for malignant diseases including lung, stomach, small intestine and liver, for melanoma and non-melanoma skin cancer, non-Hodgkin’s lymphoma (NHL) and leukaemia was noted. For non-Hodgkin’s lymphoma the risk was nearly doubled five to nine years after the diagnosis of sarcoidosis [5].

There are many theories explaining the possible relationship of this association. The changes in the number and the functions of immune cells in sarcoidosis are well described, suggesting dysfunction in the immunoregulatory pathways leading to granuloma formation. A clone may escape in an environment with defective T suppressor cells regulation resulting in the development of lymphoid malignancy. In this context, chronic sarcoidosis represents a more prolonged and more severe form of the disease. Steroid treatment, by further compromising the immune system, might be an additional predisposing factor [2]. There are some cases in which LD developed subsequent to sarcoidosis [6,7], but only a few case reports present malignancy preceding sarcoidosis. Suen *et al.* described six patients who developed malignancy first, followed by the diagnosis of sarcoidosis. The median interval between the diagnoses of the two diseases was only nine months [8]. There are also some patients with NHL, who developed sarcoidosis subsequent to the diagnosis of LD. Kornacker *et al.* reported two cases, where NHL was identified and treated for 3 and 10 months before the diagnosis of sarcoidosis. They proposed that sarcoidosis had been triggered by immunological disturbance induced by chemotherapy or associated with the lymphoma. They supposed an infectious agent, which could have spread due to the immunosuppression (caused by chemotherapy) resulting in granuloma formation. Alternatively antineoplastic therapy might have reduced suppressor T cells leading to lymphocyte activation observed in sarcoidosis [9]. Sybert *et al.* reported a patient with osteosarcoma who developed granulomatous lymphadenopathy and multiple pulmonary nodules confirmed as sarcoidosis following cessation of treatment. They suggested that immunosuppression inhibited the development of sarcoidosis, which became symptomatic when chemotherapy was terminated [10].

FDG-PET is a sensitive method for the staging of several malignancies, the underlying biological principle is based on the Warburg effect [11]. FDG is transported into cells by glucose transporter GLUT-1 and is metabolized to FDG-6-phosphate and trapped [12]. The degree of the FDG uptake depends on the number of transporters and on the metabolic rate. Physiological activity is detected in the brain, myocardium and genitourinary tract. Due to increased metabolism, malignant tissues typically demonstrate higher FDG uptake than benign lesions and normal tissues. In patients with sarcoidosis, FDG-PET could be used for monitoring the response to treatment because FDG uptake correlates with disease activity but is not useful for initial diagnosis as it could be misinterpreted as a malignancy [13].

In a study with 21 patients, Bae *et al.* found that BALT lymphomas show heterogeneous but identifiable FDG uptake on PET scans [14]. They analyzed the role of FDG-PET for staging and follow-up of patients with extranodal marginal zone mucosa (mucosa associated lymphoid tissue, MALT) lymphomas. A total of 42 patient scans and clinical information were reviewed. MALT lymphomas have high FDG avidity and PET scan is usable for detection of areas of transformation and for staging. In 34 of the 42 patients, there was FDG avidity, which demonstrated that FDG-PET scan is a possible diagnostic tool for the detection of MALT lymphoma in the majority of patients. Eleven patients who had BALT lymphoma in their lung all showed focal FDG uptake on PET scans. The authors suggested that with the advance and spread of the technology, its role in cancer - especially in MALT lymphomas - will expand and will lead to more accurate staging and better management of the disease [15].

According to the current literature, our patient had chronic sarcoidosis and had been treated with systemic steroids. The development of autoimmunity (SLE) and its treatment with cyclophosphamide induced changes in the slow growing tumour. This is in line with the theory that abnormal immune function contributes to the development of LD, and that immunosuppressive therapy may enhance it [16,17]. The development of lymphoma presented over a decade.

## Conclusions

According to previous observations, sarcoidosis predisposes for lymphoid malignancies. Sarcoidosis and lymphoma both can cause increased FDG uptake in mediastinal lymph nodes, thus FDG-PET-CT cannot prevent the need for histological verification [18]. In our patient suffering from sarcoidosis, malignancy was only confirmed by cytology so FDG-PET-CT was an important diagnostic tool in the further decision-making process. The progression of the lung lesion, later confirmed as BALT lymphoma, occured following steroid and cyclophosphamide treatment (both agents used in the treatment of lymphomas), which was retrospectively interesting and unexpected. Future studies are needed to assess the full potential of FDG-PET-CT in the diagnosis of sarcoidosis lymphoma syndrome.

## Consent

Written informed consent was obtained from the patient for publication of this case report and any accompanying images. A copy of the written consent is available for review by the Editor-in-Chief of this journal.

## Abbreviations

ANA: Antinuclear antibodies; BALT: Bronchus associated lymphoid tissue; CO: Carbon-monoxide; CT: Computed tomography; FDG: Fluorodeoxyglucose; LD: Lymphoproliferative disease; MALT: Mucosa associated lymphoid tissue; NHL: Non-Hodgkin’s lymphoma; PET: Positron emission tomography; SLE: Systemic lupus erythematosus.

## Competing interests

The authors have no competing interests to declare.

## Authors’ contributions

AK wrote the paper. NE and LT have been involved in drafting the manuscript. GYL and VM drafted the manuscript and revised it critically for important intellectual content. ACS performed the lobectomy. JCS carried out the histological examination and immunophenotyping. All authors read and approved the final manuscript.
